# Thrombopoietin Receptor Agonists in Post-Hematopoietic Cell Transplantation Complicated by Prolonged Thrombocytopenia: A Comprehensive Review

**DOI:** 10.2147/ITT.S463384

**Published:** 2024-09-13

**Authors:** Abdelrahman Elsayed, Basant Elsayed, Mohamed Elmarasi, Ahmed Adel Elsabagh, Engy Elsayed, Ibrahim Elmakaty, Mohamed Yassin

**Affiliations:** 1 Department of Medical Education, Hamad Medical Corporation, Doha, Qatar; 2 College of Medicine, Qatar University, Doha, Qatar; 3 Hematology Section, National Center for Cancer Care and Research (NCCCR), Doha, Qatar

**Keywords:** hematopoietic cell transplantation, thrombocytopenia, thrombopoietin receptor agonists, recombinant human thrombopoietin, romiplostim, eltrombopag

## Abstract

Hematopoietic cell transplantation (HCT) is a well-established procedure that has become a therapeutic mainstay for various hematological conditions. Prolonged thrombocytopenia following HCT is associated with a significant risk of morbidity and mortality, yet no universally recognized treatment protocol exists for such a complication. First-generation thrombopoietin receptor (TpoR) agonists as well as second-generation agents are known for their role in enhancing platelet production, and their use is expanding across various thrombocytopenic conditions. Therefore, we conducted this comprehensive review of the literature to provide an updated evaluation of the use of TpoR agonists and explore their efficacy and safety in the treatment of extended post-HCT thrombocytopenia. The literature search was conducted using PubMed database from 1996 through December 2023, using a predefined strategy with medical subject headings terms. We identified 64 reports on the utility of TpoR agonists, five of them were randomized controlled trials and the rest were retrospective observational studies and case series, with a total number of 1730 patients. Second-generation TpoR agonists appear more convenient than subcutaneous recombinant human thrombopoietin (rhTpo) as they can be orally administered and exhibit similar efficacy in platelet recovery, as indicated by recent trial results. Among these agents, avatrombopag, unlike eltrombopag, does not require any dietary restrictions, which could be more favorable for patients. However, eltrombopag remains the most extensively studied agent. TpoR agonists had promising effects in the treatment of post-HCT thrombocytopenia with a good safety profile so far, highlighting the potential benefit of their use.

## Introduction

Hematopoietic cell transplantation (HCT) is a well-established, multi-step procedure involving the collection of hematopoietic stem cells, conditioning the patient with a regimen before infusing the stem cells, and ultimately reestablishing a new hematopoietic and immunological system.^1^ It initially emerged in the early 1960s as a rescue treatment for cancer patients following intense chemotherapy and radiation treatments, as well as to address severe hematopoietic system deficits.^2^ Since then, it has expanded into an adoptive immunological therapy for many conditions, including malignant and non-malignant hematological conditions and autoimmune diseases.^3^ HCT is classified as autologous or allogeneic based on the source of hematopoietic cells. In autologous HCT, after receiving a preparative regimen, the patient’s hematopoietic cells taken out before undergoing intense chemotherapy are reintroduced into the patient’s body. The sources of hematopoietic cells in autologous HCT can be from a related or nonrelated donor or umbilical blood cord units.^4^

Historically, the most feared outcomes of HCT are the relapse of the underlying condition and graft-versus-host disease (GVHD). Relapse of the underlying condition is frequently the main cause of mortality within the first four years following transplantation. Beyond this time, however, there is a considerable chance that patients will experience long-term survival provided they avoid recurrence.^5^ Despite the continuous evolution of HCT practice, GVHD is still a considerable threat to all patients. A trial testing the effects of using preemptive prednisolone therapy in patients with allogeneic HCT compared to placebo did not find a significant effect on the incidence or the severity of acute graft-versus-host disease (aGVHD), which is reported to contribute by as high as 50% to non-relapse-related mortality.^6^ Multiple studies report increased rates of GVHD incidence over the years. For instance, a study aimed to analyze the trends in the incidence and outcome of chronic graft-versus-host disease (cGVHD) over 12 years reported that the incidence has increased over time, even after adjusting for factors, such as donor type, graft type, and conditioning intensity.^7^ On the other hand, many studies reported improved mortality for HCT patients. The number of individuals who have undergone autologous and allogeneic HCT and survived is steadily rising. For patients who have maintained remission during the first two to five years after transplantation, it is estimated that around 80–90% of them will still be alive during the following ten years.^5^ These findings of increased survival and decreased rates of complications have been consistent among many studies that compared earlier versus recent eras of HCT practice.^8–11^ Given the improved survival rates observed among post-HCT patients, a convergence with the general population’s lifespan underscores the urgency of addressing the associated long-term complications. A salient concern in this regard is post-transplant thrombocytopenia, a protracted condition that poses a substantial risk of morbidity for post-HCT patients. Despite the magnitude of this issue, a universally recognized treatment protocol for post-transplant thrombocytopenia has yet to be established. Thrombopoietin receptor (TpoR) agonists, including both first-generation and second-generation agents, have emerged as pivotal interventions due to their demonstrated efficacy in augmenting platelet production. The utilization of these agents is progressively expanding across diverse thrombocytopenic conditions. Consequently, we embarked on an exhaustive literature review to deliver an updated and comprehensive assessment of the use of TpoR agonists. Our aim is to explore the efficacy and safety of these agents specifically in the context of treating prolonged post-HCT thrombocytopenia.

## Materials and Methods

### Search Strategy and Selection Criteria

The review focus was centered on peer-reviewed clinical papers involving the use of first-generation TpoR agonists (recombinant human thrombopoietin [rhTpo]) and second-generation TpoR agonists (eltrombopag, romiplostim, avatrombopag, lusutrombopag, and herombopag) in patients who underwent HCT due to malignant and non-malignant hematologic conditions and had thrombocytopenia either due to poor graft function, delayed platelet engraftment, secondary failure of platelet engraftment or in few instances to test the efficacy of TpoR agonist to facilitate and speed the process of platelet engraftment. Studies were set as not eligible if it was in a foreign language, exceptions were made for a few studies about rhTpo that were reported in Chinese. Animal studies were not included.

A comprehensive search strategy was employed in the PubMed database, covering the period from 1996 to the first of November 2023, with the aim of thoroughly investigating the utilization of TpoR agonists in addressing thrombocytopenia following HCT. The predefined search strategy incorporated medical subject headings (MeSH) terms such as “Thrombopoietin”, “Hematopoietic Stem Cell Transplantation”, and “Thrombocytopenia”. The articles identified underwent assessment for eligibility by two independent reviewers, considering relevance. Subsequently, the selected articles were retrieved and subjected to full-text assessment by two reviewers independently, adhering to inclusion and exclusion criteria. Data were then extracted from the included articles and presented in tables.

### Included Articles

**Figure 1:**
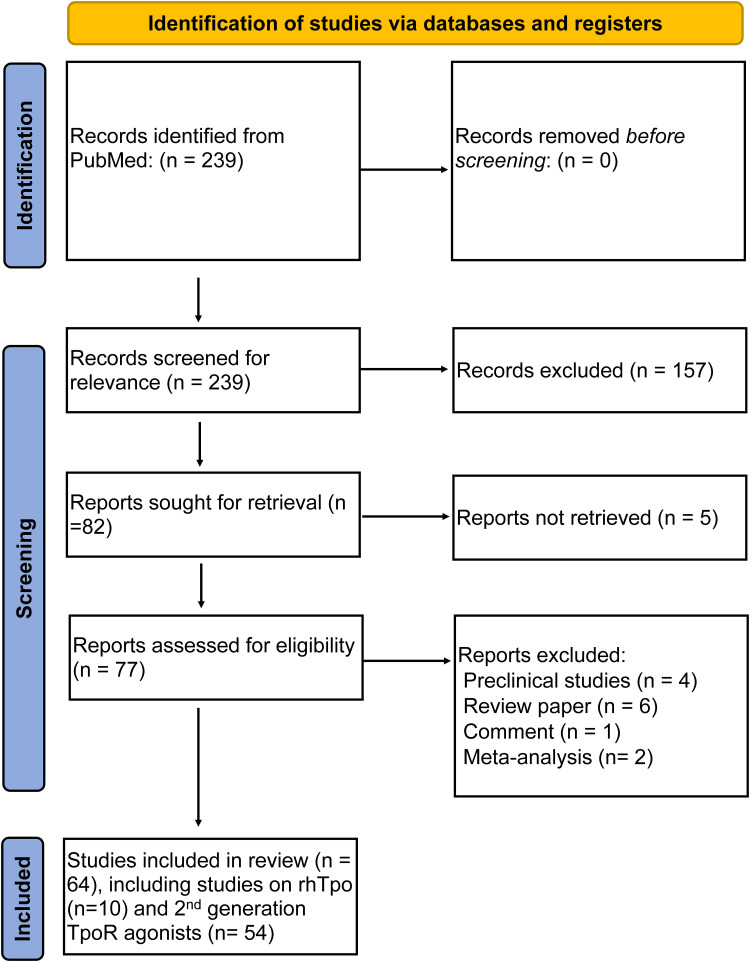
Flowchart summarizing the findings of our search strategy.

**Abbreviations**: rhTpo, recombinant human thrombopoietin; TpoR, thrombopoietin receptor.

The summary of the electronic search strategy is presented using Figure 1. Our initial search identified 239 articles that underwent title and abstract screening for relevance. Of those, only 82 articles underwent full-text retrieval and assessment, and few studies were found ineligible for inclusion and were excluded, yielding a total of 64 studies to undergo data extraction and to be summarized in two tables. The first table includes ten studies on the use of rhTpo in patients with thrombocytopenia post-HCT, covering 777 patients. Meanwhile, the second table comprises 54 studies on the utilization of second-generation TpoR agonists.

## Post-HCT Thrombocytopenia

### Pathophysiology of Idiopathic Post-HCT Thrombocytopenia

Following myeloablative conditioning and autologous stem cell rescue, all patients experience profound thrombocytopenia, which is caused by bone marrow aplasia brought on by high-dose chemotherapy and/or total body irradiation (TBI). Following autologous hematopoietic progenitor cell transplantation (HPCT), the return to normal blood leukocyte and platelet counts typically occurs within the first month after transplant, with rising blood cell counts of lymphocytes, granulocytes, platelets, and eventually erythrocytes.^12^ Post-transplant thrombocytopenia can be caused by a variety of reasons, including GVHD, infections (such as cytomegalovirus), immune-mediated factors, drug-related factors (such as ganciclovir and valganciclovir), disease recurrence, and thrombotic microangiopathy.^13^,^14^ Historically, post-transplant thrombocytopenia was categorized as either secondary failure of platelet recovery (SFPR) or prolonged isolated thrombocytopenia (PIT).^15^ PIT is defined as the need for thrombocyte suspension transfusions for more than 60 days following HCT or sufficient engraftment of all peripheral blood lineages, except for platelets, being < 20x10^9^/L. On the other hand, SFPR is defined as losing independence on platelet transfusions for seven straight days after allogeneic HCT with a number of thrombocytes < 20x10^9^/L from over 50x10^9^/L. According to the reports, PIT and SFPR occur in 12–20% and 20–40% of patients, respectively.^16^ Post-HCT thrombocytopenia and lower initial posttransplant platelets are considered poor prognostic indicators as they reflect poor engraftment, can cause bleeding, and have been associated with higher mortality rates.^12^,^17^

There are two basic theories explaining the origin of protracted isolated thrombocytopenia, while the precise etiology is unknown. Anti-platelet autoantibodies, splenic sequestration, or other conditions may cause normal platelets produced by the bone marrow to be prematurely destroyed in the peripheral circulation. Alternately, poor megakaryocyte differentiation may result in insufficient platelet production.^18^ One study measured glycocalicin index (GCI), a parameter that reflects platelet turn-over, serum thrombopoietin (Tpo) that is inversely proportional to megakaryocytes activity (ie, elevated in aplastic anemia) and circulating B-cells producing anti-GPIIb-IIIa in patients with or without thrombocytopenia post-HCT and patients with aplastic anemia, immune thrombocytopenia, and healthy individuals has found that CGI and Tpo levels in patients with post-HCT thrombocytopenia were similar to patients with aplastic anemia, suggesting a major role of impaired thrombocytosis as a cause of thrombocytopenia,^14^ with similar conclusions were reported in other study.^19^ In addition, antiplatelet antibodies were also detected in patients with thrombocytopenia proving the possible role of autoantibodies in the development of post-HCT thrombocytopenia and consistent with other findings in the literature as well.^20^ These findings conclude a complex, multifactorial pathophysiology behind persistent post-HCT thrombocytopenia that remains not fully understood until now.

### Treatment of Idiopathic Post-HCT Thrombocytopenia

Despite the advancements in the practice of HCT, definitive treatment regimens for post-HCT thrombocytopenia have not been established yet. Historically, the mainstay treatment of post-HCT thrombocytopenia was repeated platelets transfusion that has been used during the pancytopenia phase post-HCT, along with other blood products to maintain patients’ blood components.^21^ Potential morbidities associated with repetitive platelet transfusions include transfusion reactions, platelet alloimmunization, and increased costs.^22^ As a result, huge efforts were put to decrease the need for platelet transfusions and reduce bleeding complications.

### Thrombopoietin and Thrombopoietin Receptors

TpoR also known previously as C-MPL or CD110 was first identified in the early 1990s.^23^,^24^ The human TpoR is made up of various components. Its 466 amino acid extracellular portion has two cytokine receptor motifs (CRMs). In addition, there is a transmembrane section made up of 22 amino acids, followed by a cytoplasmic portion consisting of 122 amino acids.^25^ Of the two extracellular CRMs, it seems that ligand binding occurs at the motif that is furthest from the membrane which also functions as a regulator when in the unbound state, preventing the cytokine receptor motif closest to the membrane from continuously signaling.^26^

Tpo also known as C-MPL ligand, the glycoprotein composed of 332-amino acid, is the most potent cytokine that physiologically regulates platelet production, produced continuously in the liver and other organs, travels via the bloodstream to the bone marrow where it promotes the early emergence of numerous hematopoietic lineages.^27^,^28^ Tpo levels are usually inversely proportional to thrombocytes in the blood and marrow megakaryocyte abundance.^29^ Regulation of Tpo is thought to involve complex processes, one proposed mechanism is that Tpo is produced continuously and the abundance of platelets that have TpoRs binds, absorbs Tpo, and breaks it down, thus reducing Tpo levels when there is a high platelet count and when the platelet count is low, Tpo is not degraded and increases in the blood.^30^,^31^ This model explains states of marrow failure such as aplastic anemia; however, it does not explain the unexpected decrease in Tpo levels in immune thrombocytopenia observed by some studies.^32^,^33^ The theory was then adjusted after noticing elevated bone marrow megakaryocytes, which naturally express TpoRs explaining why blood Tpo is decreased in this disorder. Yet, the theory still does not explain the relationship between Tpo production and the abundance of platelets or megakaryocytes in other disorders such as reactive thrombocytosis associated with inflammation, or infection. Multiple factors that might induce increased Tpo levels have been described in the literature highlighting the complexity and multifactorial nature of Tpo regulation.^26^

## First Generation of Thrombopoietic Agents

Two molecules were developed in the early nineties that were found to have a stimulating activity on the thrombopoietic effects. The first was rhTpo, a glycosylated compound manufactured using Chinese hamster ovary (CHO) cells, encompassing the complete native human amino acid sequence. Administration of a sole rhTpo dose led to a rise in platelet count, commencing around the fifth day for the majority of patients and reaching its zenith at an average of day 12.^34^ Pegylated human recombinant megakaryocyte growth and development factor (PEG-rHuMGDF), a truncated polypeptide Mpl-ligand derivatized with poly-(ethylene glycol), was produced in E-coli and had the first 163 amino acids of the human Tpo in its sequence,^35^ an induced megakaryocyte endoreduplication and proliferation in vitro and in vivo. Changes in platelet production and function produced by PEG-rHuMGDF returned to baseline within two weeks after discontinuing treatment. Initial studies showed that PEG-rHuMGDF increases platelet production in a linear log-dose-dependent manner by stimulating megakaryocyte endoreduplication and new megakaryocyte formation from marrow hematopoietic progenitors in nonhuman primates.^36^

Although PEG-rHuMGDF initially showed promise in early clinical trials by being safe and effective at increasing platelet levels, the development of neutralizing antibodies and severe thrombocytopenia in around 8% of exposed patients due to its homology with the native Tpo led to discontinuation of trails in the United States.^37^,^38^ On the other side, similar adverse effects were not encountered with the use of rhTpo. Only a single study has reported that a patient tested positive for non-neutralizing transient antibodies to full length which was not associated with any clinical consequences.^34^ As this finding was not reported elsewhere it did not hinder the clinical development of that potentially beneficial medication that has an array of indications, particularly in China where it was approved for the treatment of chemotherapy-induced thrombocytopenia (CIT).^39–41^ The use of rhTpo for the treatment of post-HCT thrombocytopenia was investigated by many studies in the past with ongoing efforts to prove its benefits.^42^ Table 1 summarizes the available studies on the use of rhTpo for the treatment of post-HCT thrombocytopenia treatment. A cross all the reported nine studies that included more than 700 patients, rhTpo was reported to demonstrate a degree of efficacy for promoting platelet recovery after HCT and treatment of platelet engraftment failure.^42–51^ A trial including 120 patients who underwent allogeneic HCT were randomized to receive rhTpo from day-1 post-transplant revealed a statistically significant cumulative incidence of platelet engraftment was significantly higher in the rhTpo group than in the control group on day-60 post-transplantation (91.7 ± 3.8% vs 74.5 ± 5.8%, P = 0.041) as well as a significantly lower rate of delayed platelet engraftment in the RhTpo group (8.3%, 5/60) than in the control group (23.3%, 14/60) (P = 0.024).^46^ Another study included a cohort of 24 patients who underwent allogeneic HCT, 20 with DPE and four with SFPR have demonstrated a significant difference in overall response when compared to a historical group as 11 (45.8%) patients in the rhTpo group achieved platelet engraftment after 28 days of treatment versus the historical control group (12.2%, p < 0.001).^46^

**Table 1:** Summary of ten Reported Studies on the Use of rhTpo for the Treatment of Post-HCT Thrombocytopenia

Study	Study Design	Study Population	Medications	Regimen of rhTpo	Principle Findings	Adverse Effects
Nash et al, 2000^43^	Case series	Study conducted April 1996-January 1997; 38 patients enrolled, 37 evaluable; Thrombocytopenia defined as <20,000/µL; Persistence >35 days post-HCT.	rhTpo	rhTpo given as single doses of 0.6, 1.2, and 2.4 µg/kg (group A) or multiple doses every 3 days up to 5 doses (group B).	10 patients recovered platelet counts in 28 days, 3 showed increased marrow megakaryocyte content 7 days post-rhTpo treatment.	No significant adverse effects were observed.
Wolff et al, 2001^44^	Case series	33 patients underwent high dose chemotherapy followed by ABMT for breast cancer.	rhTpo	rhTpo via bolus injection at varying doses to 33 patients starting post-marrow infusion until platelet recovery (>20,000/mL), with G-CSF use.	Median platelet recovery after ABMT was 19 days (range, 11–41); rhTpo dose/schedule showed no impact on recovery; further Phase II and III trials warranted for efficacy assessment.	Serious adverse events or neutralizing antibodies to rhTpo were not observed during the study.
Lui et al, 2011^45^	Pilot study	19 patients with hematological malignancies received rhTpo pre-platelet engraftment after related donor haploidentical HCT; median age was 36 years (18–51).	rhTpo	Subcutaneous rhTpo at 1.0 mg/kg/day started on day 6 post-transplantation and continued for 14 days.	Platelet engraftment was attained by 18 patients within a median period of 16 days, ranging from 11 to 34 days.	No significant adverse effects of rhTPO were observed.
Han et al, 2015^46^	RCT	Study involved 120 patients with hematological malignancies, 60 in rhTpo group and 60 in control group.	rhTpo	15,000 U of rhTpo given daily via subcutaneous injection post-transplant.	On day 60 post-transplant, rhTpo group showed 91.7% platelet engraftment vs 74.5% in control group (P=0.041).	No severe adverse effects noted, median follow-up 256 days.
Wang et al, 2017^47^	Retrospective study	275 allo-HCT patients: 135 treated with rhTpo, 140 controls.	rhTpo	rhTpo given subcutaneously at 15,000 U daily from days +4 to +7 post-transplant.	After allo-HCT, rhTpo showed slight survival advantage overall (P = 0.159), notably significant in MDS and AA patients (P = 0.014), but not in others (acute leukemia, MPN, PNH, lymphoma) (P = 0.849).	No significant adverse effects were observed.
Song et al, 2018^48^	RCT	85 severe aplastic anemia cases underwent allo-HCT from Jan 2010 to Mar 2017, divided into rhTpo (n=29), rhIL-11 (n=27), and control (n=29) groups; median age: 35 (15–58) years.	rhTpo vs rhIL-11	rhTpo group (29 cases): initiated rhTpo 15,000 U/d subcutaneously on day +1; rhIL-11 group (27 cases): started rhIL-11 1.5 mg/d subcutaneously on day +1; control group (29 cases): no platelet-raising drugs.	No significant differences in granulocyte and platelet recovery ≥20×10(9)/L among groups (P>0.05). Time to PLT ≥50×10(9)/L shorter in rhTpo vs control (16.5 vs 22 days, P<0.05), and time to PLT ≥100×10(9)/L shorter in rhTpo (23 days) and rhIL-11 (28 days) vs control (35 days, P<0.05).	The rhTpo group had no obvious adverse events.
Sun et al, 2019^42^	Retrospective study	24 patients with prolonged isolated thrombocytopenia treated with rhTpo from July 1, 2016, to May 31, 2017.	rhTpo	rhTpo injections given at 300 IU/kg/d for 28 days or until platelet counts reached ≥ 50 × 10^9^/L, regardless of transfusion.	ORR at 45.8%, significantly surpassing historical data (12.2%, p < 0.001), with median response time of 12 (7–25) days post-rhTpo treatment initiation.	One injection site pain; 4 of 28 (14.3%) developed GVHD, and 8 of 28 (28.6%) had CMV reactivation during treatment.
Tang et al, 2020^49^	RCT	120 umbilical cord blood transplant patients randomized: treatment group median age 27 years (range: 9–59) vs control group median age 26 years (range: 8–54).	rhTpo vs none	rhTpo group received daily subcutaneous injections of rhTpo at 300 U/kg (max 15,000 U) from days 14 to 28.	rhTpo: 60-day engraftment 83.1% (95% CI, 70.3–90.7%) vs control 66.7% (95% CI, 53.0%-77.2%; P = 0.020), engraftment times: 38 vs 43 days (P = 0.237).	Discontinuation due to adverse event: injection site pain in 2 patients.
Gu et al, 2021^50^	Retrospective study	35 multiple myeloma cases enrolled in prospective trial since March 2014 compared with 98 historical cases; hematopoietic reconstitution analyzed post-stem cell reinfusion.	rhTpo	rhTpo given subcutaneously at 15,000 U daily for 14 consecutive days starting on day 1 or day 3 post-stem cell reinfusion.	Median time to neutrophil and platelet engraftment: 10th and 11th day post-stem cell reinfusion, respectively. Multivariate analysis: rhTPO independently accelerated platelet engraftment (HR 2.013, 95% CI 1.336–3.034, p = 0.001).	All the 35 patients tolerated rhTpo well.
Cao et al, 2022^51^	Case series	Retrospective analysis of 29 patients treated with continuous rhTpo for secondary failure of platelet recovery post-allogeneic HCT.	rhTpo	RhTpo administered at 300 IU/kg/day for up to 42 days or until platelet counts reached ≥50 × 10^9^/L, irrespective of transfusion.	Overall response: 24 (82.8%) patients; complete response: 10 (34.5%) patients, median time to response: 21.5 days (range: 3–41 days) and 39.5 days (range: 7–53 days) post-rhTpo initiation.	rhTpo well tolerated, no side effects requiring withdrawal or clinical intervention any patients.

**Notes**: The ten reported studies on the use of rhTpo for the treatment of post-HCT thrombocytopenia include two of which are RCTs whereas the rest are retrospective studies and case reports with a total number of 777 patients who had HCT and were treated for thrombocytopenia.

**Abbreviations**: ABMT, Autologous Bone Marrow Transplant; allo-HCT, Allogeneic Hematopoietic Stem Cell Transplantation; AA, Aplastic Anemia; CMV, Cytomegalovirus; G-CSF, Granulocyte Colony-Stimulating Factor; GVHD, Graft-versus-Host Disease; HCT, Hematopoietic Stem Cell Transplantation; MDS, Myelodysplastic Syndrome; MPN, Myeloproliferative Neoplasm; ORR, Overall Response Rate; PLT, Platelet; RCT, Randomized Controlled Trial; rhIL-11, Recombinant Human Interleukin-11; rhTpo, Recombinant Human Thrombopoietin.

Oprelvekin, a recombinant human interleukin 11 analogs that received the United States food and drug administration (FDA) approval for the prevention of CIT in patients with non-myeloid malignancies after demonstrating modest efficacy as per multiple studies in the literature.^41^,^52^ For instance, a meta-analysis demonstrated a reduction in the time needed for platelet counts to reach 50 × 10^9^/L (weighted mean difference [WMD] = −4.19 days; 95% CI: −5.01, −3.37), the time to reach 100 × 10^9^/L (WMD = −4.45 days; 95% CI: −4.85, −4.06), platelet transfusion volume (WMD = −6.14 units; 95% CI: −9.20, −3.09) in patients with acute leukemia patients with CIT.^53^ Despite the reported benefits of oprelvekin in patients with CIT, the FDA did not recommend oprelvekin following myeloablative chemotherapy due to increased rates of adverse effects such as edema, conjunctival bleeding, hypotension, and tachycardia, in addition to other fatal adverse effects reported in the post-marketing period of the drug. Few studies were conducted in China on the use of oprelvekin for the treatment of patients post-HCT have demonstrated faster platelet recovery time as compared to placebo and have also reported adverse effects associated with the use of oprelvekin.^54–56^ These adverse effects, along with the high cost of rhIL-11 led the manufacturer to discontinue producing the drug in the United States.^57^

## Second Generation of TpoR Agonists

Second-generation TpoR agonists are short peptide sequences that serve as analogs that activate TpoRs, unlike the first-generation molecules, they have no sequence homology with Tpo, therefore eliminating the dilemma of autoantibodies formation to endogenous Tpo.^58^ Late in the nineties of the last century, efforts finally discovered a 14-amino acid peptide was among the earliest identified molecules, which was able to bind to and activate the TpoR, with even higher affinity by thousands after it was dimerized.^59^,^60^ With covalently linking the tandem peptide dimers with two disulfide-bonded human IgG1 kappa light chains, the stability, and half-life of that molecule were extended, yielding the novel peptide TpoR agonist Romiplostim.^58^ Romiplostim was approved by the FDA for the treatment of immune thrombocytopenia (ITP) in adults and children aged one and above. Later, another non-peptide TpoR agonists were developed that showed efficacy for the treatment of many thrombocytopenia-related conditions such as eltrombopag that was FDA approved for many conditions such as ITP and refractory aplastic anemia, avatrombopag and lusutrombopag that are FDA approved and hetrombopag that is being under investigation currently. Efforts have been focusing on the promising benefits of these novel drugs for patients with post-HCT platelet engraftment failure and here we highlight the latest evidence for the benefits and adverse effects of utilizing these medications from this aspect. Table 2 provides a summary of all the reported studies on the utility of second-generation TpoR agonist agents.^61–114^

**Table 2:** Summary of 54 Studies Done to Assess the Efficacy and Safety of Second-Generation TpoR Agonists for the Treatment of Thrombocytopenia

Study	Study Design	Study Population	TpoR agonist	Regimen	Principle Findings	Adverse Effects
Zheng et al, 2023^61^	Prospective clinical trial	17 patients after HCT due to AML with a median age of 38 years.	Herombopag	Herombopag was orally applied (7.5 mg/day) from day +1 post-SCT.	Herombopag group had significantly higher cumulative incidence of PPE, CPE, and NE compared to control (PPE +21%, 88% vs 65%, p = 0.003; CPE +30%, 65% vs 43%, p = 0.001; NE +14%, 76% vs 53%, p <.001).	Well-tolerated and safe. Adverse effects reported are unlikely to be due to Herombopag.
Al-Mashdali et al, 2023^62^	Case series	4 patients with SFPR (n=2) and PIT (n=2) post-haploidentical HCT (n=3) and allo-HCT (n=1) for AML (n=2), AA, and DLBCL; median age 35 (range: 29–48).	Romiplostim	Romiplostim doses ranged between 4–10 µg/kg weekly. The median time to start treatment 61 (range:23–230).	All patients responded; median time to platelet count ≥50 × 10^3^/μL: 44 days (range: 4–132), ≥100 × 10^3^/μL: 104 days (range: 11–164).	No patient has developed any adverse effects.
Kırcalı et al, 2023^63^	Retrospective study	39 patients with persistent PFG-induced cytopenia post-HCT; median age 49 years (range: 18–65).	Eltrombopag	Treatment began at 12.5 mg dose, gradually reaching max dose of 150 mg/day.	84.6% responded to EPAG, 15.4% remained transfusion-dependent; 1-year OS: 75% for responders (n = 33) vs 66.7% for non-responders (n = 6, p = 0.3).	1 ended eltrombopag to venous thromboembolism; 5 patients (12.9%) had up to grade I fibrosis.
Ruan et al, 2023^64^	Retrospective pilot study	30 patients (median age 6 years) post-allo-HCT for hematological conditions compared for Avatrombopag indication in PGF, SFPR, or PIT.	Avatrombopag	Initial dose: 10 mg for <30 kg, 20 mg for ≥30 kg, increased up to 40 mg based on response; median initiation post-HCT: day +25.	ORR: 91% (platelet ≥20 × 109/L for ≥7 consecutive days without transfusion); complete response rate: 78% (platelet ≥50 × 10^9^/L for ≥7 consecutive days without transfusion).	No severe adverse events were documented with Avatrombopag.
Zhou et al, 2022^65^	Retrospective study	61 consecutive HCT patients, median age 43, developed thrombocytopenia; 35 (57.6%) DPE, 26 (42.6%) SFPR cases.	Avatrombopag	Initial dose: 20 mg daily, increased by 20 mg every 2 weeks, maximum dosage up to 60 mg/day; Avatrombopag initiated at median 48 days.	In the entire group, 42 (68.9%) of 61 patients responded to Avatrombopag with median response time 21 days (IQR 6–33); cumulative ORR: 69.1%.	No fatigue, overt thrombosis, or grade III–IV organ toxicities according to NCI-CTC in any patients.
Wen et al, 2022^66^	Non-inferiority, RCT	Patients eligible for allo-HCT for various hematologic conditions enrolled; 44 randomized to eltrombopag (median age 36) and 48 to rhTpo group (median age 37).	Eltrombopag vs rhTpo	Oral eltrombopag (50 mg daily) or subcutaneous rhTpo (15,000 U daily) initiated post-transplantation.	Eltrombopag as effective as rhTpo for platelet engraftment post allo-HCT in hematological malignancies; oral eltrombopag more convenient.	No differences in adverse events between eltrombopag and rhTpo.
Yan et al, 2022^67^	Retrospective study	34 patients, median age 35.5 years (range: 15–61), had thrombocytopenia post-haplo-PBSCT; 7 PIT, 27 SFPR.	Eltrombopag	Dose initiated at 25 mg or 50 mg daily, increased by 25 mg every 2 weeks up to max 100 mg/day; reduced by 25 mg weekly at response.	22 patients (64.7%) responded including 15 CR and 7 PR patients. PIT vs SFPR comparison: OR cumulative incidence 44.4% vs 78.3% (P = 0.0657), CR cumulative incidence 33.3% vs 52.7% (P = 0.0989).	No grade 3 or 4 toxicities observed; eltrombopag discontinued in 2 patients due to headache/nausea.
Güven et al, 2022^68^	Retrospective study	Study included 48 HCT recipients, median age 53 years (range: 21–69); 30 (62.5%) experienced DPR and 18 (37.5%) experienced SFPR.	Eltrombopag	Eltrombopag 25 mg or 50 mg, increased by 25 mg weekly up to 150 mg; median start time: 57 days (range: 36–513 days).	23 patients responded; cumulative incidence of successful platelet recovery: 48%; 38% for DPR and 50% for SFPR in allogeneic transplantation.	No drug-related side effects; no need for drug discontinuation.
Karataş et al, 2022^69^	Retrospective study	25 patients; median age: 53.3 (23.9–65) years; 60% underwent AHCT, 40% underwent allo-HCT.	Eltrombopag	Eltrombopag initiated at 25 mg/day, increased weekly by 25 mg/day up to 150 mg/day.	Eltrombopag responded in AHCT (66.7%) and allo-HCT (50%) recipients, with a significant survival duration	No adverse effects that required drug discontinuation.
Samarkandi et al, 2022^70^	Retrospective study	21 post-HCT thrombocytopenia patients, median age 27 (range: 7–58).	Eltrombopag	Eltrombopag 50 (25–150) mg daily initiated at median 91 (31–787) days post-HCT.	63% (10/16) achieved ≥20,000/μL platelet count without transfusion; 33% (7/21) reached ≥50,000/μL without transfusion.	Well tolerated; no drug discontinuation to adverse events.
Bostankolu Değirmenci et al, 2022^71^	Retrospective study	27 allo-HCT recipients with median age 55 (range: 21−73) years experienced SFPR and PGF.	Eltrombopag	Eltrombopag 25 mg daily, up to 75 (range: 50–100) initiated on day 110 (range: 33–670).	CR achieved in 16 patients (59.3%), with time-to-treatment response of 42 days (range: 3−170 days).	Fibrosis, increased with longer time to response (p = 0.008).
Bortolotti et al, 2022^72^	Case report	60-year-old with amyloidosis AL received AHCT, requiring 3 extra stem cell infusions.	Eltrombopag	Eltrombopag 50 mg/day to 100 mg/day, stopped on day 52 to limited efficacy; restarted on day 129 at 150 mg/day.	Transfusion independence achieved by day 200 for RBC and day 249 for PLT. Eltrombopag discontinued by day 536 due to stable PLT.	No adverse events observed during eltrombopag therapy.
Matsumoto et al, 2022^73^	Case report	4-year old male received allo-HCT for Diamond–Blackfan anemia with PIT complication.	Eltrombopag	Eltrombopag 1.0 mg/kg/day on post-transplant Day 124, up to 1.5 mg/kg/day on Day 137.	Platelet count remained >20 × 10^9^/L from Day +294 post-transplant, eltrombopag stopped 1 year and 10 months later.	Grade 1 hepatic function disorder and diarrhea.
Ahn et al, 2022^74^	Case series	8 patients with PGF, median age at transplant: 46 years (range: 18–73).	Eltrombopag	Median maximum dose: 50 mg/day. Median start time: 73 days (range: 23–477).	Hemoglobin increased from 8.2 g/dL to 10.9 g/dL; neutrophil count from 1.25 × 10^9^/L to 3.32 × 10^9^/L; median platelet count from 18.5 × 10^9^/L to 54 × 10^9^/L.	3 grade 2 liver enzyme elevations but resolved.
Zhu et al, 2022^75^	Retrospective study	13 patients experienced SFPR, 3 had PT after haploidentical HCT, median age 45.5 (16–58) years.	Avatrombopag and umbilical cord MSCs	Initial avatrombopag dose: 20mg/d orally for ≥2 weeks; 40mg/d if PLT low; terminated if PLT>400×10^9^/L.	16 patients: 81.3% achieved CR, 12.5% PR, and 6.3% NR; median time to CR: 32 (7–426) days post-treatment with avatrombopag and umbilical cord MSCs.	No drug discontinuation due to adverse reactions or intolerance.
Ahmed et al, 2021^76^	Prospective RCT	60 patients randomized: 42 to eltrombopag, 18 to placebo; 7 AHCT, 53 allo-HCT.	Eltrombopag vs placebo	Eltrombopag initiated at 50 mg orally daily, escalated every 2 weeks up to 75 mg, 125 mg, and 150 mg if platelet count <50,000/mL; East Asian patients started at 25 mg.	In eltrombopag arm, 15/42 patients (36%) had platelet >30,000, compared to 5/18 (28%) in placebo arm (not statistically significant). For platelet count >50,000, 9/42 (21%) in the eltrombopag arm, and 0/18 in the placebo arm (P = 0.046).	Eltrombopag arm had a statically significant higher rates of hyperbilirubinemia.
Pasvolsky et al, 2021^77^	Phase 2a single arm RCT	12 patients, including 10 adults and 2 children, median age at transplantation 50 years (range 6–74), selected for unmanipulated single or double UCB allogeneic grafts.	Eltrombopag	Oral eltrombopag until PLT>50,000/mL for 14 days; starting doses: 100 mg/day for adults, 50 mg/day for children weighing 20–40 kg, and 2 mg/kg/day for children <20 kg.	Median time to partial platelet engraftment: 55 days (range: 25–199); complete platelet engraftment: 66 days (range: 31–230);; platelets >20,000/mL and >50,000/mL: 55 days (range: 25–199) and 66 days (range: 31–230), respectively.	Treatment was safe and well tolerated even at maximal doses.
Yaman et al,2021^78^	Retrospective study	18 pediatric patients (median age: 15 years, range: 5–18) treated for SFPR (n=6) and PIT (n=12) after allo-HCT.	Eltrombopag	Starting dose: 50 mg/d (<11 years, >20 kg); 75–100 mg (12–18 years), up to 12.5 mg until platelets >50,000/μL; max dose: 75 mg/d (<11 years), 150 mg/d (older).	14 of 18 patients achieved transfusion independence within median 14 days; 77% sustained platelet count >30,000/μL in median 21 days, and 72.2% >50,000/μL in median 44 days.	None of the patients had to discontinue the drug because of adverse events or intolerability.
Giammarco et al, 2021^79^	Retrospective study	Thrombocytopenia in 94%, anemia in 77%, and neutropenia in 42% of 48 allogeneic HCT patients.	Eltrombopag	Treatment initiated 95 (range 17–877) days post-HCT, dose 25–100 mg/day.	ORR: 75%, with 24 complete responses and 12 partial responses; median time to response: 60 days (range: 14–300).	Eltrombopag well tolerated, no treatment-related adverse events reported.
Gupta et al, 2021^80^	Case report	5-year-old male with Hodgkin lymphoma underwent AHCT, complicated by DPE.	Eltrombopag	Started oral eltrombopag at 25 mg/day on day+56.	Platelet recovery initiated with treatment, reached 100 ×10^3^/cumm on day 110.	No adverse effects reported.
Uria-Oficialdegui et al, 2021^81^	Retrospective study	5 pediatric patients with poor graft function post-alloHCT; median age: 11 years (range: 8–17).	Eltrombopag	Median time from HCT to initiation: 120 days; dose: 50 mg/day, up to 75 mg/day.	3 patients (60%) achieved complete response*, 1 partial response (20%), and 1 (20%) showed no response.	All patients tolerated eltrombopag well, no adverse events.
Nampoothiri et al, 2021^82^	Retrospective study	17 patients received eltrombopag for post-Allo-HCT complications; median age at Allo-HCT: 58 years (range: 23–73).	Eltrombopag	50 or 100 mg orally daily, increased every 2–4 weeks up to 150 mg daily.	76.5% ORR (13/17), with 10/13 complete responses and 3/13 partial responses. In PGF patients (n = 15), ORR was 86.7%.	No reported adverse effects that could be attributed.
Liu et al, 2021^83^	Retrospective study	24 post-HCT patients with thrombocytopenia, median age 7.7 (2.6–13.7) years.	Eltrombopag	Total dose: 1400 (200–5900) mg; median duration post-HCT: 27.5 (8.0–125.0) days.	Complete response rate was 92% (22/24).	No eltrombopag related adverse reactions.
Qiu et al, 2021^84^	Retrospective study	43 patients with persistent thrombocytopenia post-allo-HCT; 33 (76.7%) PIT, 10 (23.3%) SFPR; median age: 6.6 (range: 2.5–14.8).	Eltrombopag	Initiated at median 27.0 days post-HCT; dose 0.5–1.5 mg/kg/d; median maintenance dose 2.1 mg/kg/d.	35 children responded to eltrombopag; cumulative PLT recovery incidence: 88.9%, 27 (77.1%) with PT, 8 (22.9%) with SFPR.	6 children experienced liver injury, but none discontinued eltrombopag.
Lancman et al, 2020^85^	Case series	Median age of 5 patients: 39 years (range: 35–41 years).	Romiplostim	Started 59 days (range, 38–396 days) post-HCT with 1 mcg/kg weekly.	80% response rate observed in 4 out of 5 patients.	One patient had bone marrow fibrosis post-treatment.
Aydin et al,2020^86^	Case series	12 patients developed refractory cytopenias post-transplant: graft failure (n = 1) or PGF (n = 11) after allo-HCT; median age: 47.5 (range: 40–66).	Eltrombopag	Initiated 214 days post-HCST; eltrombopag doses varied from 50–150 mg/day.	Induced transfusion independence in 10/12 patients, significantly raising platelet counts (from 16,000 to 77,000 × 10^6^/L, p = 0.012).	Stopped one patient for elevated LFTs and venous occlusive disease.
Masetti et al, 2020^87^	Case series	9 pediatric patients with did HCT with a median age being 14.1 years (range 0.9–18.3). Five patients (55%) had HCT from an unrelated donor and three from a haploidentical donor.	Eltrombopag	Treatment was started at a median time after HCT of 147 days (range 83–330). The starting dose being 50 mg per day.	After median treatment of 36 days, 88% reached sustained platelet count >50,000/μL; cumulative incidence of sustained platelet count >50,000/μL without transfusion support was 75% at 60 days.	One patient had grade I transaminitis.
Gao et al, 2020^88^	Retrospective study	32 patients with PGF (n=15) and SFPR (n=17) post-HCT, median age 27 (range: 14–62).	Eltrombopag and rhTpo	rhTpo given for at least 2 weeks before eltrombopag initiation. Eltrombopag started 50 mg daily, maximum 75 mg for <50 kg, 100 mg for ≥50 kg.	Overall recovery: 21 (65.6%) patients, including 7 PR and 14 CR.** No significant difference between PPGF and secondary SPGF.	All patients tolerated EPAG well without thrombosis, liver, or kidney issues.
Peffault de Latour et al, 2020^89^	Prospective trial	24 patients from 4 French hospitals, median age 43 years (range: 33.8–61.5); 10 DPR, 14 thrombocytopenia post-allogeneic HCT.	Romiplostim	Median time from HCT to romiplostim initiation: 85 days (range 42–259).	18 thrombocytopenic patients responded at 45 days (range, 21–77); Hemoglobin improved in 21 of 22.	Overall, romiplostim was considered well tolerated.
Bento et al, 2019^90^	Retrospective study	86 patients: 16 with PT, 71 with SFPR, median age 53 years (range: 8–74 years), underwent various types of HCT, primarily for hematological malignancies.	Eltrombopag (n=51) and Romiplostim (n=35)	Eltrombopag 50 mg/day (25 to 150 mg/day), max 150 mg/day, Romiplostim - 1 µg/kg/week (1 to 7 µg/kg/week) and 5 µg/kg/week (1 to 10 µg/kg/week).	Platelet recovery ORR: 72%; SFPR group: 73%, PT group: 67%; PT group took longer to respond than SFPR group (median: 93 days vs 60 days); 17/22 patients with initial neutrophil count <1000/µL reached ≥1000/µL after therapy.	Grade III–IV liver abnormalities and fatigue seen in 2% of patients.
Li et al, 2019^91^	Case series	3 pediatric allo-HCT patients: 2 with secondary thrombocytopenia, 1 with platelet engraftment failure.	rhTpo and Eltrombopag	50 mg daily; duration from transplantation to eltrombopag: 79 days (range: 50–156).	Two patients achieved platelet stabilization ≥50x10^9^/L post-eltrombopag, one failed platelet engraftment remained transfusion dependent.	No adverse effects experienced.
Fu et al, 2019^92^	Retrospective study	38 patients with refractory thrombocytopenia after haploidentical allo-HCT: 8 had DPE, 15 had SFPR, and 15 had PGF.	Eltrombopag	Initiated at 179 days post-haplo-HCT, 25 or 50 mg daily, adjusted to maintain platelets 50–100 × 10^9^/L.	24 patients responded: cumulative OR 63.2%, 6 (25.0%) DPE, 9 (37.5%) SFPR, and 9 (37.5%) PGF; median time to response 17 days (range: 2–89 days).	5 patients (13.2%) experienced liver injury unrelated to eltrombopag.
Marotta et al, 2019^93^	Case series	12 patients with PGF and 1 with primary graft failure post allo-HCT.	Eltrombopag	Initial dose: 50mg/day, with possible increases every 2–4 weeks, up to maximum of 150mg/day.	6 achieved complete response*** and 1 partial response.	No clinically meaningful side effect was observed.
Yuan et al, 2019^94^	Retrospective study	13 allo-SCT recipients evaluated: 6 with PIT, 7 with SFPR; median age 53 years (range: 36–70 years).	Eltrombopag	Initial eltrombopag dose 25 or 50 mg/day, adjusted per manufacturer’s recommendations.	Patients with both PIT and SPFR responded, with higher response rate in SPFR (68% vs 49% in PIT). 3 in PPF and 2 in SPFR did not respond to eltrombopag.	Eltrombopag treatment well tolerated, no discontinuations.
Rivera et al, 2019^95^	Retrospective study	14 patients post-allo-HCT, median age 56 years (range: 25–69).	Eltrombopag	Eltrombopag initiated at 50 mg once daily, escalated weekly to max 150 mg/day.	8 patients achieved platelet recovery after a median of 91 days (range: 8–206); 7 had SFPR and 1 had PIT.	No thrombosis or grade III–IV toxicities reported.
Tang et al, 2018^96^	Retrospective study	12 patients with PGF after HCT.	Eltrombopag	Eltrombopag initiated at 25 mg/d for 3 days, then increased to 50 or 75 mg/d.	ORR: 83.3% (10/12); 8 patients achieved CR in all cell lines with median time to CR of 29 days (range: 10–49).	Eltrombopag well tolerated by all 12 patients.
Bosch-Vilaseca et al, 2018^97^	Retrospective study	Study included 20 adult allo-SCT recipients, median age 48 years, 90% had SFPR, 2 had PFPR.	Romiplostim and Eltrombopag	Eltrombopag: 50 mg/day; Romiplostim: 2 μg/kg weekly, max doses: 150 mg/day eltrombopag, 10 μg/kg weekly Romiplostim; TpoR agonists started median 150 days post-allo-SCT (range: 50–1041).	Romiplostim: 18 (90%) patients, Eltrombopag: 2 (10%) patients; Overall response: 12/20 (60%); 180-day cumulative incidence of platelet recovery to ≥30 x 10^9/L: 57% (95% C.I.: 44–71%) reached at median of 28 days from therapy start.	TpoR agonists well tolerated; only 2 patients showed possibly related adverse effects (grade III liver disease, fatigue).
Master et al, 2018^98^	Case report	26-year-old female with AML had delayed engraftment post-allo-HCT.	Eltrombopag	Eltrombopag was started at 50 mg orally daily on day 72.	All 3 cell lines responded at 1 year neutrophil count from 1,000/µL to 8,000/µL, hemoglobin from 8 g/dL to 14 g/dL, and platelets from 20,000/µL to 120,000/µL.	The patient tolerated eltrombopag well.
Hartranft et al, 2017^99^	Retrospective study	13 patients received romiplostim post-allo-HCT, 9 with SFPR, 4 with platelet engraftment failure. Median age: 48 years (range: 17–68 years).	Romiplostim	Romiplostim initiated at median dose 1.5 mcg/kg weekly, titrated to max 10 mg/kg/dose; started median 91 days post-HCT.	54% response rate to treatment with median response time of 35 days (range 14–56); responders had median survival of 753 days post-HCT vs 266 days for non-responders (P = 0.0375).	No adverse effects of Romiplostim were reported.
Ali et al, 2017^100^	Case report	10-year-old boy with severe aplastic anemia underwent allogeneic HCT complicated by SFPR.	Eltrombopag	Starting dose: 25 mg (0.5 mg/kg/d) daily; increased to 50 mg daily after 2 months.	Platelet counts stabilized above 10×10^9^/L after 2 months; treatment stopped after 10.7 months.	No side effects reported.
Tanaka et al, 2016^101^	Case series	12 patients, median age 53 years (range: 19–63), 7 with SFPR, 5 with PIT post HCT.	Eltrombopag	Eltrombopag started at 12.5 mg daily, increased weekly until PLT>50,000/mL; median start time post-HCT: 5.6 months (range: 2.1–59.5).	Cumulative incidence of successful platelet recovery: 72%. 73% of transfusion-dependent patients became independent, with median duration to last transfusion: 8 days (range: 0–61 days).	Well tolerated, and no patients discontinued the drug because of adverse events.
Maximova et al, 2015^102^	Case series	7 pediatric patients with SFPR post-HCT, median age 11 years (range: 8–13 years).	Romiplostim	Median time from start of RS: day +85 (range 62–104) post-HCT.	All patients except one (patient 4) achieved platelet transfusion independence by the second week of treatment.	No discontinuation of drug because of adverse events.
Raut et al,2015^103^	Case series	12 adult patients, median age 29.5 years, received AHCT or allo-HCT; 11 had primary thrombocytopenia post-HCT due to lack of platelet transfusion donors.	Eltrombopag	25 mg once daily, started post-infusion: 21 days (range: +17 - +60), median treatment duration: 29 days.	8 patients achieved platelet value above 30,000/cmm.	No dose limiting toxicities have been observed.
Fujimi et al, 2015^104^	Case report	55-year-old female with stage IVA follicular lymphoma experienced failed platelet engraftment and PIT after allo-PBSCT; tested positive for Anti-TPOR antibodies at day +258.	Romiplostim and eltrombopag	Romiplostim started at 1 μg/kg on day +280, escalated weekly to 10 μg/kg based on platelet counts. Eltrombopag began on day +443 at 12.5 mg.	Romiplostim ineffective for 20 weeks; after starting eltrombopag, platelet counts increased steadily, becoming transfusion-independent by day +513.	No adverse effects from treatment with either medication.
Battipaglia et al, 2015^105^	Case series	8 patients underwent allo-HCT with SFPR (n=3) and grade III–IV hemorrhagic complications (n=5), median age at transplant 53 years (range 16–67).	Romiplostim	Starting dose: mostly 3 μg/kg weekly, with exceptions; dose escalation based on platelet count and response.	All SFPR patients achieved platelet recovery in median 2 doses (range: 1–5); median time to hemorrhage recovery for 3 evaluable patients: 44 days (range: 9–106).	2 died by transplant-related complications; no needed drug discontinuations.
Buchbinder et al, 2015^106^	Case report	5-year-old Hispanic female with graft failure post-unrelated donor umbilical cord blood transplantation for Fanconi anemia.	Romiplostim, erythropoietin and G-CSF.	Romiplostim: 10 µg/kg/week, erythropoietin: 150 units/kg/dose (3 times per week), G-CSF: 5 µg/kg/day.	By Day +130, neutrophil count >0.5x 10^9^/L and platelet count >25x 10^9^/L; by Day +149, growth factors stopped, cytopenias resolved without transfusion.	No side effects or toxicities reported.
Buchbinder et al, 2014^107^	Case report	3-year-old Asian male with symptomatic SFPR after HCT for X-linked chronic granulomatous disease from a matched unrelated female donor.	Romiplostim	Starting dose: 5 µg/kg subcutaneous weekly.	Platelet count >50,000/µL within 3 weeks of starting romiplostim, then dose tapered to maintain count >50,000/µL; total treatment duration 6 months.	No toxicities or adverse effects experienced.
DeRemer et al, 2013^108^	Case report	59-year-old male received non-myeloablative HCT for B-cell chronic lymphocytic leukemia from matched, unrelated donor.	Romiplostim	Started day +202 after steroid and IVIG failure, initially at 1 µg/kg/week, escalated to max dose of 10 µg/kg.	Persistent thrombocytopenia (<20x 10^9^/L) and transfusion dependent; day +228 biopsy showed no relapse but decreased trilineage hematopoiesis.	Testing for anti-TPO or anti-romiplostim antibodies was negative.
Poon et al, 2013^109^	Case series	3 patients with post allo-HCT PGF (n=2) and SFPR (n=1), median age: 57 years (range: 36–62).	Romiplostim	Case 1 and 3: 2 μg/kg every 2 weeks, Case 2: started at 5 μg/kg, then 10 μg/kg.	Case 1: Platelet count rose after 11 days, achieved transfusion independence within 2 weeks. Case 2: Response after 4 weeks, platelet count exceeded 100,000/μL. Case 3: Platelet count surpassed 50,000/μL after 18 weeks.	No adverse effects reported and no increase in bone marrow fibrosis.
Bollag et al, 2012^110^	Case report	48-year-old woman with SFPR post-allo-HCT for CML.	Romiplostim	The starting dose was 1 µg/kg with increments of 1 µg/kg weekly.	Romiplostim paused after peak count of 216 x 10^9^/L, restarted at 7 µg/kg weekly after drop to 26 x 10^9^/L, maintaining stable platelets at 90–200 x 10^9^/L.	No adverse effects reported.
Reid et al, 2012^111^	Case series	Case 1: 63-year-old female with allo-HCT for AML and PGF. Case 2: 42-year-old female with AHCT for AML and DPE.	Eltrombopag	Case 1: Treatment started on day +140 at 50 mg/day. Case 2: Treatment initiated after 14 months at 50 mg/day.	Case 1: Platelet transfusion independence in 2 weeks, platelets reached 30,000/uL. Case 2: Response in 2 weeks, remained transfusion independent.	No adverse effects related to drug reported. Case 1 died due to relapse.
Calmettes et al, 2011^112^	Case series	7 patients with SFPR post-allo-HCT, median age: 57 years (range: 25–60).	Romiplostim	1 µg/kg, increased weekly by 1 µg/kg until PLT reached 50,000/µL, discontinuation if PLT ≥50,000/µL.	All patients’ thrombocytopenia corrected, median time to platelet count of 50,000/mL: 54 days (range: 24–84), median treatment duration: 13 weeks (range: 4–16).	Romiplostim well tolerated; no treatment discontinuations.
Gangatharan and Cooney, 2011^113^	Case report	58-year-old man with HIV, experiencing DPE post-AHCT for acute myelogenous leukemia.	Romiplostim	Romiplostim initiated at day 80, 375 mcg weekly.	PLT 420x10^9^/L by day 111, romiplostim reduced and withdrawn after 21 doses, with platelet count maintained > 100x10^9^/L.	No adverse effects reported.
Beck et al, 2010^114^	Case report	4-year-old male underwent allo-HCT for adrenoleukodystrophy with ITP complication.	Romiplostim	Dosing: Started at 1 mcg/kg, increased to 3 mcg/kg over four weekly subcutaneous doses.	PLT increased from <10 to 30 x 10^9^/L after second week, reaching 49 x 10^9/L after fourth dose; two weeks later, count rose to 150 x 10^9/L, remaining above 100 x 10^9^/L.	No adverse effects reported.

**Notes**: The 54 studies done to assess the efficacy and safety of second-generation TpoR agonists for the treatment of thrombocytopenia include 3 prospective clinical trials, and the rest are retrospective studies and case series with a total number of 953 patients who had HCT. * CR was defined as a sustained platelet count ≥ 50 × 10^9^/L, Hb ≥ 100 g/L, and ANC ≥ 1.5 × 10^9^/L without transfusions and growth factors support for ≥7 consecutive days. **CR was defined as platelet count ≥ 100 × 10^9^/L for at least seven consecutive days. PR was defined as platelet count ≥ 50 × 10^9^/L but less than 100 ×10^9^/L for at least seven consecutive days without transfusion. ***CR was defined as all the following: i. platelet count > 80,000/μL; ii. hemoglobin >110 g/L; iii. absolute neutrophil count >1,500/μL.

**Abbreviations**: AA, Aplastic Anemia; AHCT, Autologous Hematopoietic Cell Transplantation; AML, Acute Myeloid Leukemia; Anti-TpoR, Anti-Thrombopoietin Receptor; CML, Chronic myeloid leukemia; CPE, complete platelet engraftment; CR, Complete Response; DLBCL, Diffuse Large B-Cell Lymphoma; DPE, Delayed Platelet Engraftment; EPAG, Eltrombopag; G-CSF, Granulocyte Colony-Stimulating Factor; HCST, Hematopoietic Stem Cell Transplantation; HCT, Hematopoietic Cell Transplantation; IQR, Interquartile Range; ITP, Immune thrombocytopenia; IVIG, Intravenous Immunoglobulin; LFTs, Liver Function Tests; MSCs, Mesenchymal Stem Cells; NCI-CTC, National Cancer Institute Common Terminology Criteria for Adverse Events; NE, Neutrophil Engraftment; ORR, Overall Response Rate; OS, Overall Survival; PBSCT, Peripheral Blood Stem Cell Transplantation; PFPR, Primary Failure of Platelet Recovery; PGF, Primary Graft Failure; PIT, prolonged isolated thrombocytopenia; PLT, Platelets; PPE, Post-Transplantation Erythrocytosis; PR, Partial Response; PT, Primary Thrombocytopenia; RBC, Red Blood Cells; RCT, Randomized Controlled Trial; rhTpo, Recombinant Human Thrombopoietin; SFPR, Secondary Failure of Platelet Recovery; TpoR, Thrombopoietin Receptor.

### Romiplostim (AMG 531, Nplate^®^, Romiplate^®^)

Since 1997, the primitive structure of the first molecule that had no sequence homology with Tpo started developing as an anticipated substitute for first-generation TpoR agonists.^59^ Romiplostim, a peptide TpoR agonist that’s structure was achieved by linking four of the 14-amino-acid peptides to the C-terminus of an IgG1 Fc fragment creating a peptibody. Using polyglycine linkers, two of the four 14-amino-acid peptides were attached to the Fc-gamma chain. The peptibody has functional constant domains (CH2 and CH3) that can bind to the FcRn receptors which internalize it and then release it back into the circulation by exocytosis thus explaining its prolonged half-life.^115^

Although it is believed that romiplostim has lower affinity than native Tpo, it produces an immediate activation cascade starting with phosphorylation of the receptor and initiation of the JAK2 and STAT5 pathways stimulating CFU-Mk growth and increased megakaryocyte ploidy in a dose-dependent fashion.^116^ It was approved by the FDA in 2008 for ITP and administered weekly as a subcutaneous injection at a dose of 1 to 10 mcg/kg and also approved for patients with radiation injury-related thrombocytopenia. In addition romiplostim has been tested for patients with chemotherapy-induced thrombocytopenia,^117–120^ myelodysplastic syndrome with thrombocytopenia,^121–123^ perioperative thrombocytopenia,^124^,^125^ in approved in Japan for thrombocytopenia in aplastic anemia after studies have shown benefit for its use there.^126^,^127^

The utility of romiplostim use in patients with thrombocytopenia post-HCT has been questioned in multiple case reports and retrospective case series reported in Table 2.^108^,^110^,^113^,^114^ The only available prospective trial in this context reported that 18 patients achieved a sustained platelet (>50x 10^9^/L) of 24 patients with delayed platelet engraftment (n = 10) and secondary thrombocytopenia (n = 14) due to either GVHD or infection. In addition, a hemoglobin response was also observed in 21 of 22 patients and a response in neutrophil counts in four patients who had <1000x 10^9^/L before treatment initiation.^89^ However, spontaneous recovery could not be excluded as the cause of these effects because the study lacked a control group. Another retrospective study included 13 pediatric patients with SFPR (n = 9) and engraftment failure (n = 4) reported platelet transfusion independence after one week of treatment with romiplostim despite a platelet count of <10x10^9^/L before treatment initiation.^102^ As with other TpoR agonists, the response to romiplostim seems to correlate inversely with low bone marrow functionality reflected by low megakaryocyte counts on bone marrow biopsies, which is thought to explain the cause of resistance to the treatment of romiplostim in some of the reported cases.^19^,^108^

### Eltrombopag (SB497115, Promacta^®^, Revolade^®^)

Eltrombopag is the first non-peptide Tpo agonist identified by using screening strategies of chemical libraries.^128^ Interestingly, many compounds were found to display bioactivity on TpoRs, Hydrazone compounds were described as thrombopoietin receptor mimetics as they were found to be activating STAT proteins in a Tpo responsive cell line.^129^ As a member of the bioarylhydrazone class of chemical compounds, eltrombopag has a metal chelate group in the center with an acidic (COOH) group at one end and lipophilic (CH3) groups at the other end.^115^ In vitro eltrombopag is a highly potent activator of the STAT and MAPK signaling pathways and induces the differentiation of bone marrow precursor cells and proliferation of Tpo-dependent cell lines. In cells expressing other factors other than TpoRs, eltrombopag did not show activity on these cascades highlighting that the activation of JAK/STAT by eltrombopag is due to the specific activation of the Tpo.^108^ Similar to all nonpeptide TpoR agonists eltrombopag binds to the transmembrane region of the TpoR at the histidine 499 amino acid, therefore it does not compete with Tpo for the binding site and its effects seem to be additive to Tpo in various in vitro experiments.^130^

In 2008, eltrombopag was initially approved as the first oral agent to increase platelet count and to be used for rare blood disorders, mainly ITP, and later its utility has been expanding after many clinical trials proved its efficacy.^131^ These approvals were based on evidence from trials on conditions such as aplastic anemia,^132–134^ and thrombocytopenia due to Hepatitis C-related cirrhosis.^135^,^136^ Among the TpoR agonists, eltrombopag is the most studied agent in the context of post-HCT thrombocytopenia with 24 reports of case series and retrospective studies and three published clinical trials including 687 patients (Table 2), as well as few case reports. Collectively, eltrombopag has demonstrated efficacy in raising platelet counts for patients with SFPR or PIT post HCT and decreasing platelet transfusion dependence. A recent trial including 60 patients randomized to receive eltrombopag or placebo demonstrated a statistically significant difference in the rates of achieving platelet count 50,000/µL (the study’s secondary endpoint) despite having a nonsignificant difference between the eltrombopag group and the placebo group at a threshold of 30,000/µL (the study’s primary endpoint) and therefore highlighting the efficacy of eltrombopag in raising platelet counts aside from the spontaneous recovery of platelets.^76^

On the other side, a prospective single-armed trial analyzed the efficacy of eltrombopag treatment from day one after transplantation until platelet count exceeded 50,000/µL for 14 consecutive days in 12 patients undergoing allogeneic transplantation compared to a historical cohort that concluded no statistically significant between the two groups in regards to partial and complete platelet engraftment.^77^ Therefore questioning the need for routine administration of anti-TpoR agents for patients undergoing HCT. Eltrombopag was shown to be effective for the treatment of SFPR and DPR, a study on a total of 86 adult patients post HCT, 16 with PT and 71 with SFPR reported overall response for platelet recovery was 72%, including 73% in the SFPR group and 67% in the PT group without a statistical significance between the groups and a longer time to response for patients with PT compared with those with SFPR with a median of 93 days and 60 days respectively.^90^ Another retrospective study on 46 patients who underwent HCT reported that 23 patients responded to treatment and the cumulative incidence of successful platelet recovery was 48%. The cumulative incidences of platelet recovery were 38% among patients with DPR and 50% among those with SFPR for allogeneic transplantation, respectively.^68^

Although eltrombopag displayed consistent safety and tolerability in the majority of the studies, few studies reported adverse events that required discontinuation of the drug. Only a single study reported that one of 39 adult patients who received treatment for PGF developed two episodes of grade II deep vein thrombosis.^63^ Eltrombopag was also associated with transaminitis, a case series on eight adult patients with PGF reported three events of grade two liver enzyme elevations were reported but resolved with conservative treatment.^74^ Another study on 43 pediatric patients with PIT and SFPR reported liver injury occurred in six children (14%) where transaminases were 2.5 times higher than the normal value or the bilirubin level was twice the normal value, however, treatment was continued.^84^ Similarly another study reported that five patients out of 38 had similar events.^92^ Hyperbilirubinemia (grade III) was also reported in six out of 24 patients and was significantly higher in the treatment group of a recent trial.^76^ However, a study reported that metabolites of eltrombopag interact with the laboratory measurements of bilirubin and therefore cause inaccurate bilirubin measurements.^137^

### Avatrombopag (E5501, AKR-50, YM-477, Doptelet^®^)

Avatrombopag is a newer non-peptide Tpo memetic and three times more potent than eltrombopag in raising platelet counts.^138^ Like other non-peptide agents, it has similar pharmacological properties such as being orally available and its binding site at the transmembrane domain that does not compete with endogenous Tpo at its distal domain binding site. The combination of avatrombopag with Tpo increased the number of differentiations of CD34+ to megakaryocytes to about 200% of that generated with Tpo only reflecting the additive properties with endogenous Tpo and its effect to induce differentiation of human stem cells.^139^ A phase-I clinical trial revealed that avatrombopag caused platelet count to increase after 3–5 days post-administration, with maximum changes of >370 × 10^9^/L over baseline with 20 mg daily after 13–16 days in healthy individuals demonstrating that its effect on platelet counts depended on dose, concentration and treatment duration.^131^ Avatrombopag induces proliferation of human TpoR-expressing murine Ba/F3 cells in a concentration-dependent manner in in vitro experiments with a maximum activity similar to that of rhTpo activating the downstream signaling cascade through tyrosine phosphorylation of STAT3 and STAT5, and threonine phosphorylation of MAPK (ERK) activating PI3K/AKT and MAPK signaling pathways.^140^ Similar to eltrombopag, without TpoR no response was noted.^141^

Avatrombopag was the first drug to be approved for the treatment of thrombocytopenia in cirrhosis patients undergoing elective procedures by the FDA in 2018. Approval was based on large phase II and III clinical trials assessing its clinical utility in this patient population that showed both efficacy and safety.^142^,^143^ Further studies have demonstrated similar efficacy of avatrombopag. A phase-II clinical trial on Japanese patients with CLD and thrombocytopenia have illustrated a significant improve in platelet counts as compared to placebo with no marked adverse effects.^144^ In 2019, the usage of avatrombopag has been expanded to include patients with thrombocytopenia due to ITP that failed to improve with other treatments as clinical trials provided evidence of the overall benefits of avatrombopag which was tolerated and effective for the treatment of chronic ITP.^145^,^146^ Recent post-hoc analysis on Phase III trial data by Jurczak et al, 2018 has demonstrated consistent conclusions and highlighted the high durability of response to treatment.^147^ Moreover, a study on patients with ITP treated with romiplostim or eltrombopag showed that patients achieved very high response rates even in those without initial response following switching to avatrombopag which illustrated the value of switching between TpoR agonists when prior TpoR agonists do not provide adequate effectiveness, convenience, or tolerability and potency of avatrombopag.^148^ In addition, avatrombopag, when compared to other TpoR agents, has practical oral dosing with a single pill strength as compared to rhTpo, has lower hepatoxicity rates, and does not require dietary restrictions in opposition to eltrombopag that requires to be only taken one to two hours before food for good absorption of the drug and to obtain a baseline liver function tests for starting the medication.^149^

So far, the utility of avatrombopag for post-HCT thrombocytopenia has been tested in three recent studies including 104 patients only where significant findings emerged. The first study, conducted on 61 patients, highlighted avatrombopag effectiveness, revealing an overall response rate of 68.9% and a complete response rate of 39.3% in patients with SFPR and DPE. Notably, the presence of adequate megakaryocytes prior to treatment was associated with a significantly higher likelihood of achieving both overall and complete responses.^65^ The second study involving 16 patients focused on avatrombopag combined with mesenchymal stem cells (MSCs). This combination exhibited promising outcomes with 81.3% achieving a complete response, suggesting that this regimen could expedite platelet recovery after transplantation.^75^ The third study, conducted on 30 pediatric patients with SFPR, PGF, and for promoting engraftment of platelets post-HCT, revealed a notably higher overall response rate (91%) and complete response rate (78%). This study identified different patient groups, distinguishing their response rates based on graft function. Specifically, avatrombopag was significantly more effective in the engraftment-promotion group compared to the PGF/SFPR recovery group. Risk factors such as severe GVHD and inadequate megakaryocytes were associated with reduced complete response rates.^64^ Importantly, all three studies reported avatrombopag as a well tolerated and potentially effective treatment option for post-HCT thrombocytopenia in adults and children, although the combination with MSCs in one study showed promise but also a severe adverse event leading to mortality most likely related to cytomegalovirus infection.

### Lusutrombopag (S-888711, Mulpleta^®^)

Lusutrombopag was initially identified by Shionogi & Company, Limited, as a non-peptide second-generation TpoR agonist that shares similar pharmacological properties as other non-peptide TpoR agonists such as being orally bioavailable and acting on the transmembrane domain of human TpoRs found in megakaryocytes.^150^,^151^ A Trial of multiple studies was conducted to evaluate the impact of food and calcium carbonate on the pharmacokinetics of lusutrombopag in healthy subjects. The results from 48 patients indicated that lusutrombopag exposure remained largely unchanged, with only a minor decrease when taken with food, and there was no significant effect when coadministered with calcium carbonate, suggesting that lusutrombopag administration does not require specific restrictions regarding meals or mineral supplements, unlike eltrombopag.^152^ Initial and recent trials have demonstrated the efficacy of lusutrombopag for the treatment of cirrhosis Child-Pugh class A and B patients with thrombocytopenia who are planned to undergo surgical or dental procedures.^153^ Newer trials have addressed cirrhosis patients with Child-Pugh Class C and thrombocytopenia. The analysis of data from multiple studies demonstrated that lusutrombopag effectively increased platelet counts in these patients and was found to be safe and well tolerated, with no treatment-related serious adverse events.^154^,^155^ Lusutrombopag was initially approved by the FDA for the treatment of cirrhosis patients with thrombocytopenia who are planned to undergo surgical or dental procedures as well as for patients with ITP. Given the novelty of the drug, its benefits and safety for patients with post-HCT thrombocytopenia or compared to other TpoR agonists have not been examined so far in the literature, although theoretically, it might be a promising option in the future.

### Herombopag (Hengqu^®^)

Herombopag is a second-generation nonpeptide TpoR agonist that was developed first by Jiangsu Hengrui pharmaceutical by structural modification of eltrombopag to improve potency.^156^ Herombopag has the same mechanism of action as eltrombopag but with greater potency as evidenced by both in vitro and in vivo experiments.^157^,^158^ According to the national medical products administration, herombopag has received it is first approval in China as a treatment for chronic ITP that is refractory to immunotherapy and as a conditional approval for the treatment of aplastic anemia.

As a promising treatment option for post-HCT thrombocytopenia, only a single pilot study has tested herombopag for its efficacy in 17 patients who had platelet engraftment failure after HCT as part of acute myelogenous leukemia (AML) population, compared to a matched historical cohort of patients.^61^ Results from this study showed a statistically significant higher incidence of partial platelet engraftment (PPE – A platelet count exceeding 20,000/μL for seven consecutive days without transfusion) and complete platelet engraftment (CPE – A platelet count exceeding 50,000/μL for seven consecutive days without transfusion) in the herombopag group as compared to placebo group with a median time to PPE and CPE of 13 days (range 8–24), 20 days (range 14–45), respectively. In addition to the demonstrated efficacy, herombopag was well tolerated among the patients with no grade IV adverse effects reported.

## Safety and Adverse Events

The safety profile of recombinant rhTpo in treating post-HSCT thrombocytopenia has been questioned across multiple studies. In Nash et al’s 2000 study involving 37 patients, no significant adverse effects were observed, establishing an initial safety baseline for rhTpo. A similar finding was stated by Wolff et al, who reported no serious adverse events or neutralizing antibodies in 33 breast cancer patients treated with rhTpo following high-dose chemotherapy and autologous bone marrow transplantation (ABMT). Subsequent studies, including Lui et al and Han et al, echoed these results, with no significant adverse effects reported in 19 and 120 patients, respectively. Additionally, Wang et al and Song et al both noted the absence of severe adverse events, further reinforcing rhTpo’s safety. Minor adverse events were occasionally reported, such as grade I pain at the injection site (Sun et al, 2019) and localized pain leading to treatment discontinuation in two patients (Tang et al, 2020). Overall, the collective data suggest that rhTpo is generally well tolerated with minimal safety concerns in the context of post-HSCT thrombocytopenia.

Second-generation TpoR agonists, including Eltrombopag, Romiplostim, and Avatrombopag, exhibit varied safety profiles in the management of post-HSCT thrombocytopenia. Eltrombopag has been the most extensively studied with consistent findings supporting its safety and efficacy. Pasvolsky et al conducted a phase 2a trial involving 12 patients, demonstrating a median time to platelet engraftment of 66 days without significant toxicity or dose reductions, even at maximal doses. Yaman et al observed no treatment discontinuations due to adverse events among 18 pediatric patients, highlighting its well-tolerated nature in younger populations. Similarly, Giammarco et al and Gupta et al reported favorable safety profiles with Eltrombopag, noting minimal adverse effects and effective platelet recovery in their respective studies. Although most of the studies reported no significant concerns regarding the safety of eltrombopag, few studies reported adverse effects that in most of the times did not results in treatment discontinuation such as transaminitis,^73^,^74^,^84^,^87^,^90^,^92^,^97^ hyperbilirubinemia,^76^,^92^ thromboembolism, and bone marrow fibrosis.^85^ Kırcalı et al, 2023 for instance, reported that one patient discontinued eltrombopag treatment because of two consecutive venous thromboembolisms and reported that mild bone marrow fibrosis developed in five patients that was however clinically insignificant. Another study reported that the longer the duration of treatment response, the higher the grade of bone marrow fibrosis was noticed.^71^ Other nonspecific symptoms such as nausea and vomiting have been reported in few studies.^67^ Given the current data, these agents generally appear safe and effective in clinical use, supporting their role in managing post-HSCT thrombocytopenia.

## Limitations

Despite the promising safety profiles and potential efficacy observed with recombinant thrombopoietin agonists (rhTpo) such as romiplostim, as well as second-generation TpoRas like eltrombopag in treating post-HSCT thrombocytopenia, the existing studies have several notable limitations. Studies investigating rhTpo and second-generation agents frequently suffer from small sample sizes and a lack of control groups, which restricts the applicability of their findings across broader patient populations. Similarly, research on second-generation TpoRas, as highlighted in studies by Nampoothiri et al and Qiu et al, often relies on retrospective analyses involving diverse patient cohorts and varying dosing regimens and till now very few randomized controlled trials have been published. This heterogeneity in study designs, patient demographics and the absence of reported effect sizes complicates direct comparisons and meta-analyses, potentially hiding nuanced safety and efficacy outcomes.

In addition, given the complicated clinical course and the comorbid conditions of most of the patients who undergo HSCT, even the reported adverse effects can be hardly attributed to the use of TPO-Ras. Moreover, the scarcity of long-term safety data for both rhTpo and second-generation TpoRas raises concerns about the emergence of unforeseen adverse events, such as bone marrow fibrosis. These collective limitations emphasize the critical necessity for large-scale, well-designed prospective trials with standardized protocols. Such studies are essential to definitively assess the efficacy, safety profiles, and optimal clinical use of rhTpo and even the newer second-generation TpoRas in the management of post-HSCT thrombocytopenia, which is currently being rarely reported on few occasions that are mostly case reports and case series.

## Conclusion

**Figure 2:**
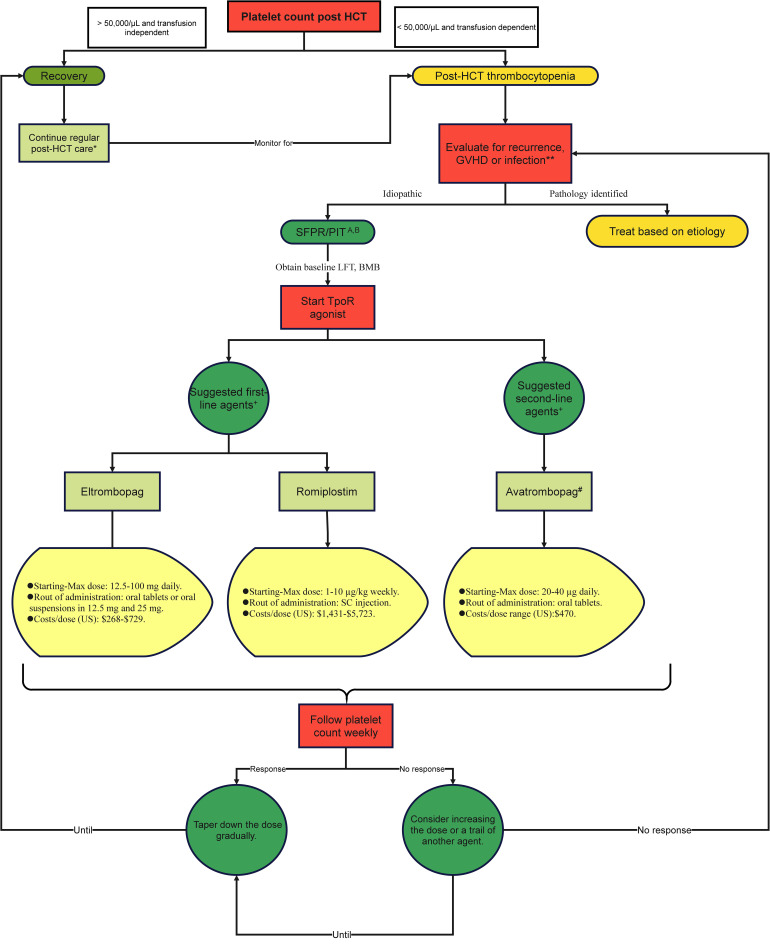
Flowchart illustrating our suggestions for second-generation TpoR agonists use in post-HCT thrombocytopenia. ^A^ SFPR definition: losing Independence on platelet transfusions for seven straight days after allogeneic HCT with a number of thrombocytes < 20x109/L from over 50x109/L. PIT definition: the need for thrombocyte suspension transfusions for more than 60 days following HCT or sufficient engraftment of all peripheral blood lineages, except for platelets, being < 20x109/L. ^B^There is currently no evidence supporting the early use of TpoR agonists to accelerate the recovery of platelet counts. * Regular individualized follow-up including screening, prevention, and counseling for all patients based on the recommendations from the guidelines. ** Investigations are based on clinical suspicion and may include laboratory work-up and histologic confirmation. + Suggested based on the level of evidence from the literature, taking into consideration the availability, costs and pharmacological properties of different agents. ^#^Does not require dietary restriction (versus eltrombopag which is only taken one to two hours before food for good absorption).

**Abbreviation**: HCT, hematopoietic cell transplantation; TpoR, thrombopoietin receptor; GVHD, graft versus host disease; SFPR, secondary failure of platelet recovery; PIT, prolonged isolated thrombocytopenia; LFT, liver function tests; BMB, bone marrow transplant; SC, subcutaneous.

Clinical studies of second-generation TpoR agonists, including romiplostim, eltrombopag, avatrombopag, and herombopag demonstrated promising effectiveness in promoting platelet recovery post-HCT, albeit with variations in response rates. Second-generation TpoR agonists seem to be more convenient than subcutaneous rhTpo as they are orally administered with a reasonable platelet recovery as. Figure 2 shows a simplified flowchart for the suggested use of second-generation TpoR agonists in post-HCT thrombocytopenia. Nevertheless, the overall efficacy and safety of TpoR agonist agents post-HCT necessitate further exploration through larger, well-structured clinical trials to optimize their use and ensure their place in standard clinical practice.

## Abbreviations

aGVHD, acute graft-versus-host disease; AML, acute myelogenous leukemia; cGVHD, chronic graft-versus-host disease; CHO, Chinese hamster ovary; CIT, chemotherapy-induced thrombocytopenia; CPE, complete platelet engraftment; CRMs, cytokine receptor motifs; FDA, food and drug administration; GCI, glycocalicin index; GVHD, graft-versus-host disease; HCT, hematopoietic cell transplantation; HPCT, hematopoietic progenitor cell transplantation; ITP, immune thrombocytopenia; MeSH, medical subject headings; MSCs, mesenchymal stem cells; PEG-rHuMGDF, Pegylated human recombinant megakaryocyte growth and development factor; PIT, prolonged isolated thrombocytopenia; PPE, partial platelet engraftment; rhTpo, recombinant human thrombopoietin; SFPR, secondary failure of platelet recovery; TBI, total body irradiation; Tpo, thrombopoietin; TpoR, thrombopoietin receptor; TpoR, thrombopoietin receptor; WMD, weighted mean difference.
